# Supplementary material for: Microbial growth and carbon use efficiency show seasonal responses in a multifactorial climate change experiment
Source: Commun Biol. 2020 Oct 16;3:584. doi: 10.1038/s42003-020-01317-1 (PMC7567817; doi:10.1038/s42003-020-01317-1)
Supplement: Supplementary file 1 — Supplementary Information [file 42003_2020_1317_MOESM1_ESM.pdf]

Supplementary Material

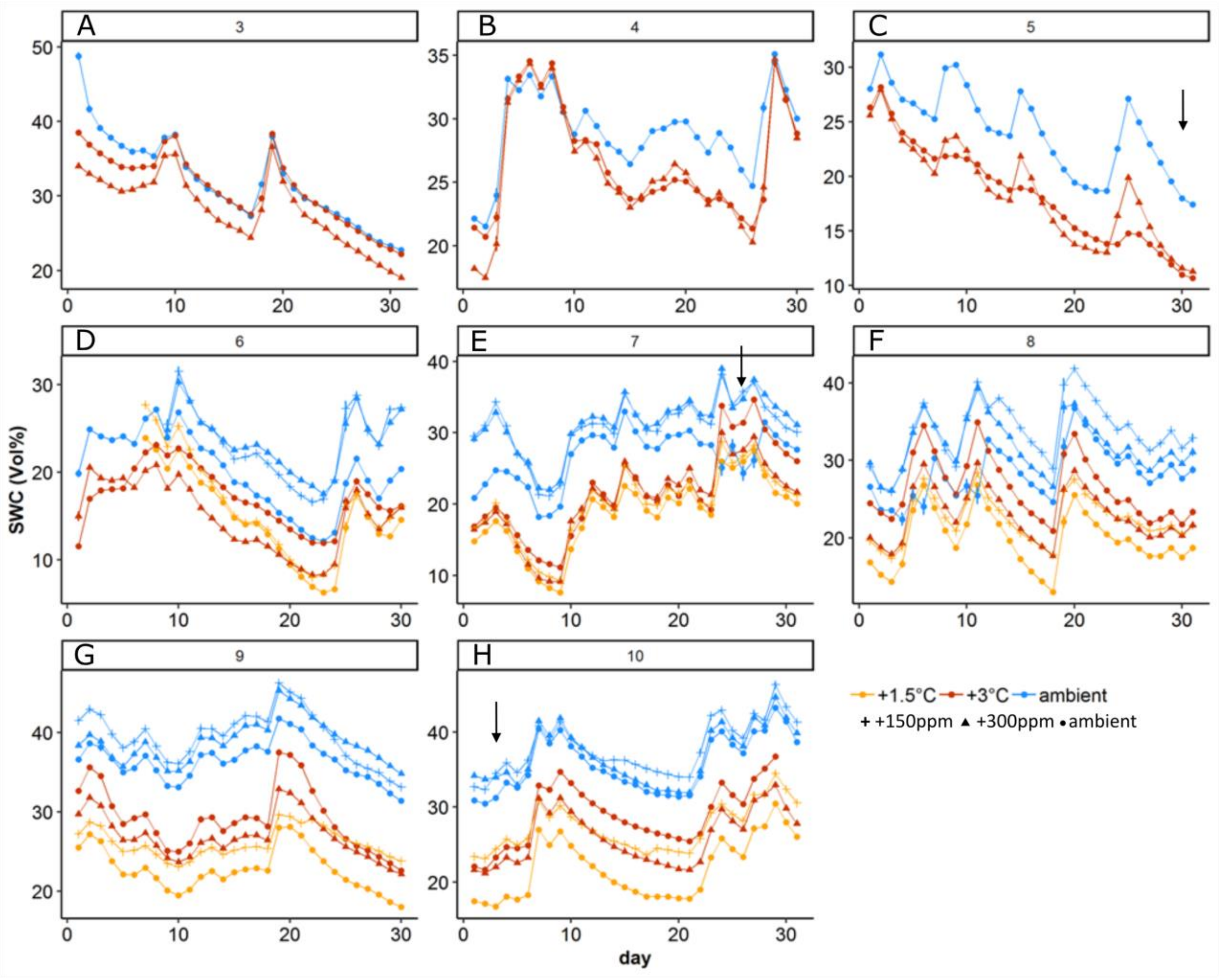

Figure S1 | **Soil water content under single and combined elevated temperature and CO_2 concentration.** Volumetric soil water content (SWC), averaged between 3 and 9 cm depth, in March **(A)**, April **(B)**, May **(C)**, June **(D)**, July **(E)**, August **(F)**, September **(G)** and October **(H)** under various combinations of three levels of temperature: ambient (sky blue), 1.5°C above ambient air temperature (orange), 3°C above ambient air temperature (red) and three levels of atmospheric CO_2 concentration: ambient (circular symbols), 150 ppm above ambient (plus symbol), 300 ppm above ambient (triangular symbols). Black arrows indicate sampling time points. Vol% = volumetric %.

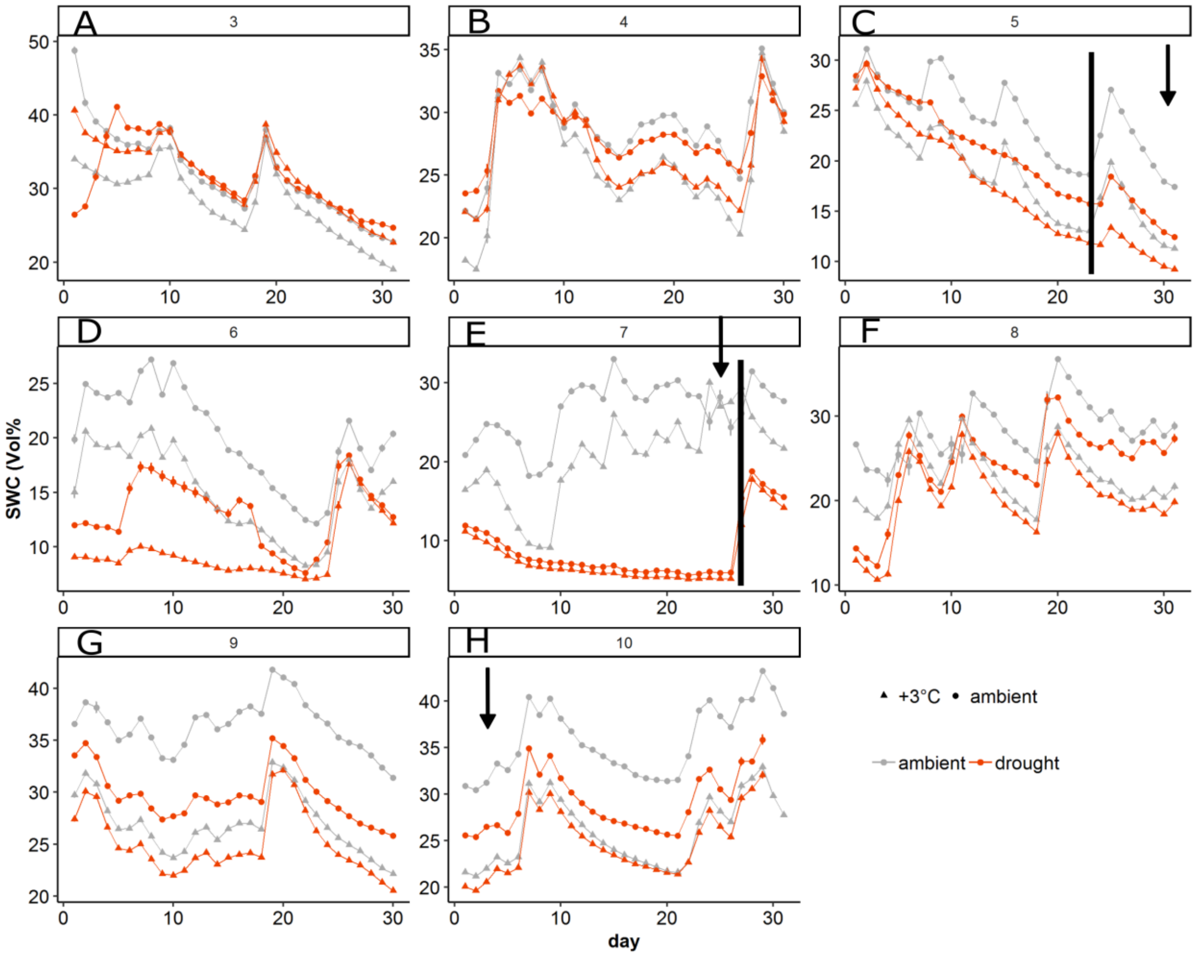

Figure S2 | **Soil water content before -, during drought and after rewetting.** Volumetric soil water content (SWC), averaged between 3 and 9 cm depth, of ambient plots (circular symbols) and future climate plots (triangular symbol, +3 °C, +300 ppm CO_2), which were exposed to drought (red) or were left unmanipulated (grey) in March **(A)**, April **(B)**, May **(C)**, June **(D)**, July **(E)**, August **(F)**, September **(G)** and October **(H)**. First vertical line represents launch of rain-out shelters, second line indicates rewetting of the drought plots, black arrows indicate sampling time points. Vol% = volumetric %.

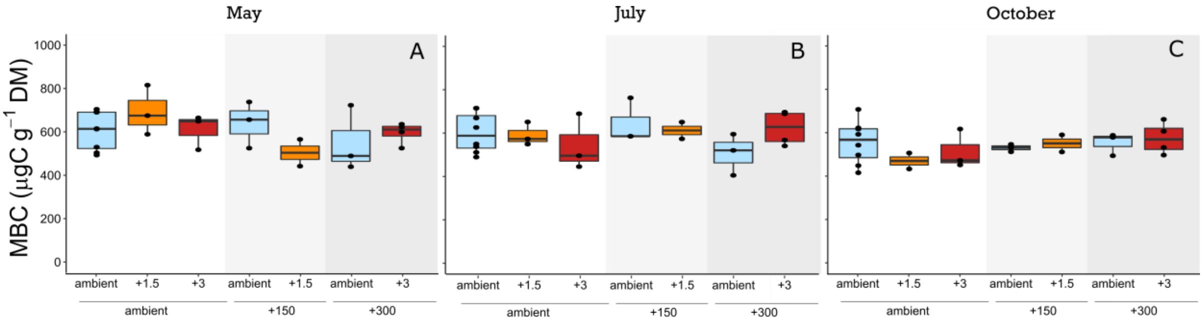

Figure S3 | **Microbial biomass across different climate change treatments.** Microbial biomass carbon per gram dry soil (MBC, µg C g^{-1} DM) at the three sampling dates: May **(A)**, July **(B)** and October **(C)** under various combinations of three temperature and three CO_2 levels: ambient air temperature (ambient, blue), 1.5 °C warmed (+1.5, orange), 3 °C above ambient air temperature heated (+3, red); ambient atmospheric CO_2 concentration (ambient, white), 150 ppm above current levels (+150, light grey), 300 ppm above current levels (+300, dark grey). Box center line represents median, box limits the upper and lower quartiles, whiskers the 1.5x interquartile range, while separated points represents outliers. (*n* = 26 of independent samples in each month, for specific replicate number of each treatment see Methods, Fig. 5)

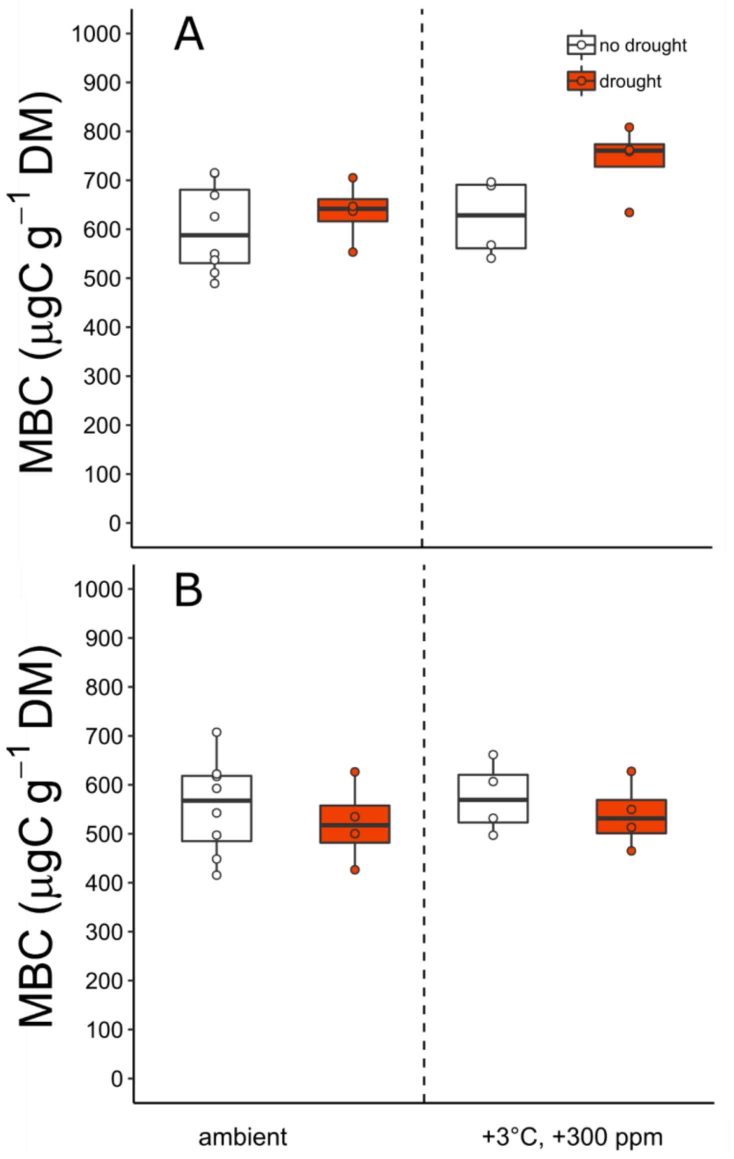

Figure S4 | **Microbial biomass during drought and two months after rewetting.** Microbial biomass carbon (MBC, µg C g^{-1} DM) in drought (red) and non-drought (white) plots **(A)**, microbial biomass (MBC, µgC g^{-1} DM) in former drought (red) and in former non-drought (white) plots 2-months after rewetting **(B)**, under ambient and future climate conditions (+3 °C, +300 ppm). DM = dry mass. Box center line represents median, box limits the upper and lower quartiles, whiskers the 1.5x interquartile range, while separated points represents outliers. (*n* = 20 of independent samples in each month, for specific replicate number of each treatment see Methods, Fig. 5)

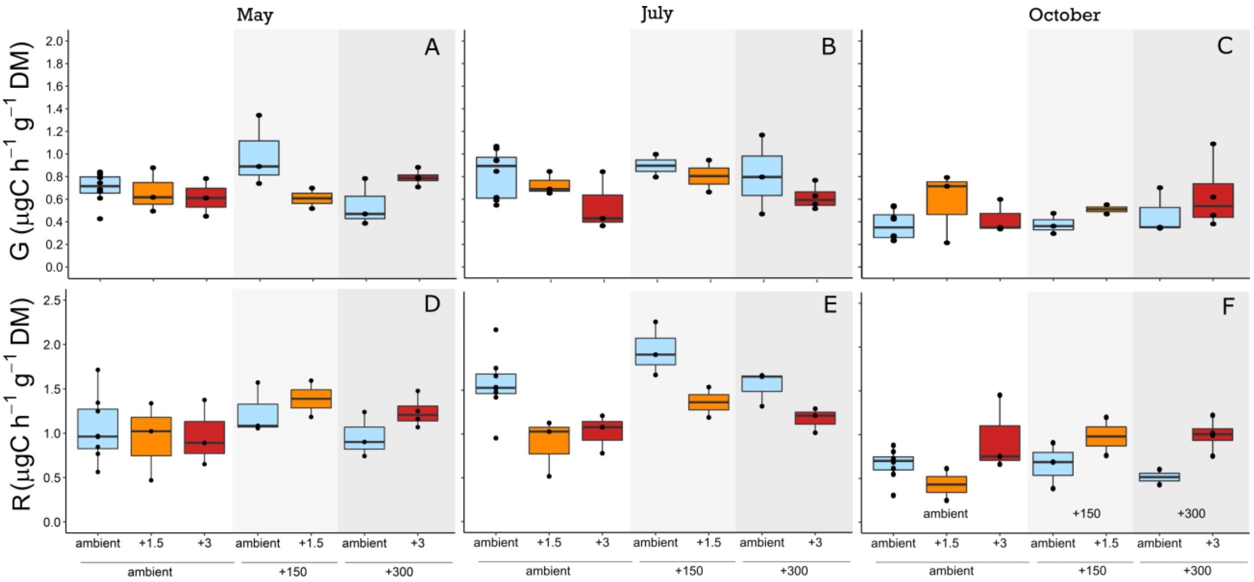

Figure S5 | **Response of microbial growth and respiration to single and combined elevated temperatures and CO_2 concentrations.** Growth per gram soil (G, µg C h^{-1} g^{-1} DM) in May **(A)**, July **(B)** and October **(C)** and respiration per gram soil (R, µg C h^{-1} g^{-1} DM) in May **(D)**, July **(E)** and October **(F)** under various combinations of three temperature - and three CO_2 levels: ambient air temperature (ambient, blue), 1.5 °C warmed (+1.5, orange), 3 °C above ambient air temperature (+3, red); ambient atmospheric CO_2 concentration (ambient, white), 150 ppm CO_2 above ambient levels (+150, light grey), additional 300 ppm CO_2 (+300, dark grey). DM = dry mass. Box center line represents median, box limits the upper and lower quartiles, whiskers the 1.5x interquartile range, while separated points represent outliers. (*n* = 26 of independent samples in each month, for specific replicate number of each treatment see Methods, Fig. 5)

Supplementary Tables

Table S1 | **Number of replicates (replicated plots in the surface response design) of the nine selected treatments and identification of treatments used in the two different statistical models tested.**

Treatment	Number of plots	Response surface model	Anova model
ambient climate	8	Yes	Yes
ambient CO_2, +1.5°C	3	Yes	No
ambient CO_2, +3°C	3	Yes	No
+150 ppm, ambient temperature	3	Yes	No
+150 ppm, +1.5°C	2	Yes	No
+300 ppm, ambient temperature	3	Yes	No
future climate (+300 ppm, +3°C)	4	Yes	Yes
ambient climate + drought	4	No	Yes
future climate + drought	4	No	Yes

Table S2 | **Incubation temperatures according to field temperature treatments.**

Temperature treatment	May	July	October
ambient	16	17	13
+1.5 °C	17.5	18.5	14.5
+3 °C	19	20	16

Incubation temperatures were set according to mean field temperatures of the three temperature treatments in May, July and October 2017.

Table S3 | **Effects of climate change treatments on microbial biomass carbon (per unit of soil mass) throughout the vegetation period.**

	May				July				October			
**MBC**	*Est.*	*SE*	*t*	*p*	*Est.*	*SE*	*t*	*p*	*Est.*	*SE*	*t*	*p*
eCO_2	-27.20	23.36	-1.16	0.2561	-3.07	22.16	-0.13	0.8909	17.16	18.43	0.93	0.3623
eT	6.51	23.60	0.27	0.7853	3.02	22.39	0.13	0.8938	-7.56	18.35	-0.41	0.6843
eCO_2:eT												
eCO_2^2												
eT^2												

Significances of elevated atmospheric CO_2 level (eCO_2) and increased air temperature (eT) as predictors of microbial biomass carbon (MBC, μgC g^{-1} DM) at each sampling time point (May, July and October). Values are derived from RSM models. eCO_2^2 & eT^2 – quadratic function of elevated CO_2 and temperature, eCO_2:T interaction of elevated CO_2 concentration and temperature. Est. = Estimated slope, SE = Standard error.

Table S4 | **Microbial biomass carbon (per unit of soil mass) during drought and two months after rewetting.**

	df	*SS*	*MS*	*F*	*p*
**MBC**					
Drought					
eCO_2 + eT	1	23076	23075.6	3.47	0.081
drought	1	22730	22729.6	3.42	0.0831
(eCO_2 + eT): drought	1	7942	7942.5	1.19	0.2907
Rewetting					
eCO_2 + eT	1	725	724.8	0.09	0.7586
drought	1	5479	5479.5	0.74	0.4026
(eCO_2 + eT): drought	1	5	4.9	0.0007	0.9799

Statistical significances of drought (drought) and climate change treatment (eCO_2 + eT), respectively significances of legacy effect of former drought plots (drought) and future climate plots (eCO_2 + eT) as explanatory variables of microbial biomass carbon (MBC, μgC g^{-1} DM) 2-months after rewetting. MBC = microbial biomass carbon, eCO_2 = elevated CO_2, et = elevated temperature. Values are derived from a two-way ANOVA. (eCO_2 + eT): drought interaction between climate treatment and drought. df = degree of freedom, SS = Sum of Squares, MS =Mean Squares.

Table S5 | **Effects of treatment and seasonality on microbial growth and respiration.**

	May				July				October			
**G**	*Est.*	*SE*	*t*	*p*	*Est.*	*SE*	*t*	*p*	*Est.*	*SE*	*t*	*p*
eCO_2	0.29	0.26	1.11	0.2798	0.01	0.05	0.28	0.7803	0.05	0.05	1.08	0.2896
eT	-0.35	0.22	-1.61	0.1237	-0.12	0.05	**-2.565**	**0.01804**	0.08	0.05	1.71	0.1011
eCO_2:eT	0.07	0.05	1.39	0.179								
eCO_2^2	-0.18	0.14	-1.33	0.1983								
eT^2	0.16	0.11	1.36	0.189								
**R**												
eCO_2	0.07	0.07	0.94	0.3573	0.08	0.39	1.95	0.6652	0.02	0.05	0.36	0.7222
eT	0.03	0.07	0.43	0.666	-1.07	0.33	**-3.27**	**0.0041**	0.14	0.05	**2.66**	**0.0149**
eCO_2:eT					0.04	0.08	0.53	0.5987				
eCO_2^2					-0.38	0.20	-1.87	0.0764				
eT^2					0.40	0.17	**2.34**	**0.0309**				

Statistical significances of the effect of (combined) elevated CO_2 level (eCO_2) and increased temperature (eT) on growth rate per gram soil (G, µgC h^{-1} g^{-1} DM) and respiration rate per gram soil (R, µgC h^{-1} g^{-1} DM). Values are derived from RSM models. eCO_2^2 & eT^2 quadratic functions, ":" indicate interaction of two or three predictors. Est. = Estimated slope, SE = Standard error, p-value < 0.05 are given in bold.

Table S6 | **Relevance of seasonality for growth and respiration.**

	G			R		
	df	*F*	*p*	*df*	*F*	*p*
date	1	**21.95**	**<0.0001**	1	**12.93**	**0.0006**
eCO_2	1	0.87	0.3549	1	3.58	0.0626
eT	1	0.03	0.8561	1	0.03	0.8688
eCO_2^2						
eT^2						
date:eCO_2	1	1.07	0.3041	1	0.35	0.5551
date:eT	1	1.51	0.2239	1	0.17	0.6827
eCO_2:eT	1	1.30	0.2585	1	0.80	0.3756
date:eCO_2:eT	1	0.58	0.4471	1	0.26	0.6119

Statistical significances of the effect of seasonality (date), elevated CO_2 level (eCO_2) and increased temperature (eT) on growth rate per gram soil (G, µgC h^{-1} g^{-1} DM) and respiration rate per gram soil (R, µgC h^{-1} g^{-1} DM), derived from GLS models. eCO_2^2 & eT^2 – quadratic functions, ":" indicate interaction of two or three predictors. df = degree of freedom, statistically significant values are given in bold (if p-value < 0.05).
