# Supplementary material for: Microbial growth and carbon use efficiency show seasonal responses in a multifactorial climate change experiment
Source: Commun Biol. 2020 Oct 16;3:584. doi: 10.1038/s42003-020-01317-1 (PMC7567817; doi:10.1038/s42003-020-01317-1)
Supplement: Supplementary file 2 — Description of Additional Supplementary Files [file 42003_2020_1317_MOESM2_ESM.pdf]

Description of Additional Supplementary Files

File Name: Supplementary Data 1
Description: Source data for the study
