# Supplementary material for: Microbial growth and carbon use efficiency show seasonal responses in a multifactorial climate change experiment
Source: Commun Biol. 2020 Oct 16;3:584. doi: 10.1038/s42003-020-01317-1 (PMC7567817; doi:10.1038/s42003-020-01317-1)
Supplement: Supplementary file 4 — Reporting Summary [file 42003_2020_1317_MOESM4_ESM.pdf]

nature research

Corresponding author(s): Alberto Canarini

Last updated by author(s): Aug 29, 2020

Reporting Summary

Nature Research wishes to improve the reproducibility of the work that we publish. This form provides structure for consistency and transparency in reporting. For further information on Nature Research policies, see Authors & Referees and the Editorial Policy Checklist .

Statistics

For all statistical analyses, confirm that the following items are present in the figure legend, table legend, main text, or Methods section.

n/a	Confirmed	
☐	☒	The exact sample size (*n*) for each experimental group/condition, given as a discrete number and unit of measurement
☐	☒	A statement on whether measurements were taken from distinct samples or whether the same sample was measured repeatedly
☐	☒	The statistical test(s) used AND whether they are one- or two-sided<br>*Only common tests should be described solely by name; describe more complex techniques in the Methods section.*
☐	☒	A description of all covariates tested
☐	☒	A description of any assumptions or corrections, such as tests of normality and adjustment for multiple comparisons
☐	☒	A full description of the statistical parameters including central tendency (e.g. means) or other basic estimates (e.g. regression coefficient) AND variation (e.g. standard deviation) or associated estimates of uncertainty (e.g. confidence intervals)
☐	☒	For null hypothesis testing, the test statistic (e.g. *F*, *t*, *r*) with confidence intervals, effect sizes, degrees of freedom and *P* value noted<br>*Give P values as exact values whenever suitable.*
☒	☐	For Bayesian analysis, information on the choice of priors and Markov chain Monte Carlo settings
☒	☐	For hierarchical and complex designs, identification of the appropriate level for tests and full reporting of outcomes
☒	☐	Estimates of effect sizes (e.g. Cohen's *d*, Pearson's *r*), indicating how they were calculated

Our web collection on statistics for biologists contains articles on many of the points above.

Software and code

Policy information about availability of computer code

Data collection	*Provide a description of all commercial, open source and custom code used to collect the data in this study, specifying the version used OR state that no software was used.*
Data analysis	The R software (version 3.4.2) was used for all statistical analyses and figures using the 'gls' function in the nlme package for the GLS models (and the function 'anova' to generate p values), the 'rsm' function in the rsm package for the response surface models, the function 'aov' for the ANOVA models and the package ggplot2 for the figures.

For manuscripts utilizing custom algorithms or software that are central to the research but not yet described in published literature, software must be made available to editors/reviewers. We strongly encourage code deposition in a community repository (e.g. GitHub). See the Nature Research guidelines for submitting code & software for further information.

Data

Policy information about availability of data

All manuscripts must include a data availability statement. This statement should provide the following information, where applicable:

- Accession codes, unique identifiers, or web links for publicly available datasets
- A list of figures that have associated raw data
- A description of any restrictions on data availability

The authors declare that the data supporting the findings of this study are available within the supplementary information files, under the name "Supplementary Data 1".

Field-specific reporting

Please select the one below that is the best fit for your research. If you are not sure, read the appropriate sections before making your selection.

☐ Life sciences ☐ Behavioural & social sciences ☒ Ecological, evolutionary & environmental sciences

For a reference copy of the document with all sections, see nature.com/documents/nr-reporting-summary-flat.pdf

Ecological, evolutionary & environmental sciences study design

All studies must disclose on these points even when the disclosure is negative.

Study description	This field experiment explored the effects of climate change drivers on microbial physiological parameters in soil sampled at three different time points during the plant growing season, in a managed montane grassland in Austria. The experimental design (the number of replicates per treatment) was based on a response surface regression approach for the warming and CO2 treatments and was combined with a factorial design testing for drought effects under ambient versus future climate (+3°C warming, +300 ppm CO2) conditions. A total of 34 plots where used for this study. At the site we manipulated 3 levels of temperature (ambient, +1.5 °C, +3°C) and atmospheric CO2 concentrations (ambient, +150 ppm, +300 ppm), and the site is additionally provided with automated rain-out shelters to simulate summer drought (see Fig. 5 for specific number of sample per each treatment). We sampled in May, July and October. Every sample was brought to the lab and was analysed for microbial biomass carbon, microbial growth, soil respiration and then we calculated the soil microbial carbon use efficiency.
Research sample	Topsoil samples were collected from grassland plots at each time point. Three to eleven soil cores (10 cm deep, 2 cm diameter) were collected at each of the 34 plots and passed through a 2 mm sieve. The topsoil was chosen since it is the most active soil horizon that is responsible for the majority of microbial activity and associated biogeochemical cycling, and will be most affected by climate change.
Sampling strategy	The plots used are part of a larger experiment tesing effects of climate change on a managed grassland and these type of experiments often involve manipulation of one or several quantitative treatment factors of interest. Response surface regression is the method of choice for these types of experiment. At this site, the experimental design involved two quantitative treatment factors, that is, elevated temperature and CO2 enrichment, and one categorical which is drought. The design strategy takes account of budget constraints imposing limitations on the number of plots with elevated temperature and CO2 levels, minimizing the number of replicate necessary. The approach is based on polynomial regression models and is focussed on an efficient estimation of interaction between the two treatment factors. Previous reported analysis demonstrated the overall suitability of the proposed design to analyse non-linear interactions of two global change factors (Piepho et al., 2017). The drought treatment was nested within the response surface design and was treated as a classic ANOVA design (n=4 per each treatment except n=8 for control plots). We collected samples at three time points during the growing season to better capture interactions between climate change effects and seasonal dynamics.<br><br>Reference:<br>Piepho, Herndl, M., Pötsch, E. M. & Bahn, M. Designing an experiment with quantitative treatment factors to study the effects of climate change. J. Agron. Crop Sci. 203, 584–592 (2017).
Data collection	Data was recorded automatically by the machines used and downloaded as an excel sheet.<br>All measurements were done and data collected by Eva Simon together with technicians.
Timing and spatial scale	Samples were collected at three time points during the growing season in 2017: in spring (30th and 31st of May), midsummer (25th and 26th of July) and beginning of autumn (3rd and 4th of October). Samples were sieved and transported to the university of Vienna, and incubated at their respective field temperatures measured at the time of harvest. Laboratory analysis were carried in the two days following arrival at the University of Vienna. The samples were collected in the central square meter of plots of 4x4 meters in size.
Data exclusions	All data available was used for the analysis. During the analysis, some outliers were detected in the RSM and ANOVA models (none in the GLS model) and they were excluded on the base of their distribution and influence on the overall model based on Cook's d test, only if they were still considered outliers after data transformation.
Reproducibility	Standard approaches, materials, machines, and methodology were used, permitting reproduction. Due to the scale of our experiment<br>and the quantity of samples and analyses required, it was impossible to repeat.
Randomization	The experimental deisgn is based on a completely randomized design.
Blinding	Blinding was not relevant to our study.

Did the study involve field work? ☒ Yes ☐ No

Field work, collection and transport

Field conditions	This study was conducted within the scope of a multifactorial climate change experiment (named ClimGrass) at the Agricultural Research and Education Centre in Raumberg-Gumpenstein, which manages the site and collects metereological data (mean annual temperature: 7.2 °C; mean annual precipitation: 1000 mm)
Location	The study site is located in Styria, Austria (47°29'37''N, 14°06'0''E), 710 meters above sea level and was established in a managed sub-montane grassland.
Access and import/export	All plots were accessible by car/walking and we were accompanied by local scientists or employees working at the Agricultural Research and Education Centre in Raumberg-Gumpenstein, which manages the site. Soil samples were all collected within Austria and no permits were necessary for transportation.
Disturbance	When the experiment was constructed, disturbance was not avoidable. In order to reduce possible confounding effects of the equipments installed to simulate warming and elevated CO2, the same equipments were installed on all plots (including control plots) and activated only where necessary. Also, the site represents a managed agricultural grassland, which is often subjected to disturbance. To reduce disturbance on the plots at the time of sampling only one person was allowed to collect soil samples.

Reporting for specific materials, systems and methods

We require information from authors about some types of materials, experimental systems and methods used in many studies. Here, indicate whether each material, system or method listed is relevant to your study. If you are not sure if a list item applies to your research, read the appropriate section before selecting a response.

Materials & experimental systems

n/a	Involved in the study	
☒	☐	Antibodies
☒	☐	Eukaryotic cell lines
☒	☐	Palaeontology
☐	☒	Animals and other organisms
☒	☐	Human research participants
☒	☐	Clinical data

Methods

n/a	Involved in the study	
☒	☐	ChIP-seq
☒	☐	Flow cytometry
☒	☐	MRI-based neuroimaging

Animals and other organisms

Policy information about studies involving animals; ARRIVE guidelines recommended for reporting animal research

Laboratory animals	This study did not involve laboratory animals.
Wild animals	Wild animals were not used in this study.
Field-collected samples	Field-collected soil samples were immediately transported to the University of Vienna and stored in airtight plastic bags for transportation. Samples were stored for about a week in incubators at the respective field temperature at the time of harvest. After completion of all analyses, soil samples were air-dried and stored at room temperature in the dark.
Ethics oversight	No ethical approval or guidance was required. We did not work with dangerous nor foreign materials (i.e. exotic species, pathogens, etc.).

Note that full information on the approval of the study protocol must also be provided in the manuscript.
